# Recurrent Postoperative Spinal Epidural Hematoma in a Patient with Protein S Deficiency

**DOI:** 10.1155/2015/536592

**Published:** 2015-07-07

**Authors:** Masato Anno, Takashi Yamazaki, Nobuhiro Hara, Keishi Hayakawa

**Affiliations:** Department of Orthopaedic Surgery, Musashino Red Cross Hospital, 1-26-1 Kyonan-cho, Musashino-shi, Tokyo 1800023, Japan

## Abstract

A 71-year-old man underwent cervical laminectomy and developed two symptomatic epidural hematomas during the acute postoperative period. On both occasions, drain obstruction was the predominant cause. Congenital Protein S deficiency was diagnosed postoperatively. Protein S is a vitamin K-dependent natural anticoagulant and is essential for inhibiting thrombosis in microcirculation. We assume that Protein S deficiency followed by perioperative bed-rest and surgical invasiveness led to severe hypercoagulability and subsequent drain obstruction. The present findings suggest that both bleeding disorders and hypercoagulability are risk factors for postoperative symptomatic epidural hematoma.

## 1. Introduction

Neurologic compromise due to postoperative spinal epidural hematoma (PSEH) is a rare but devastating complication. Only one case of recurrent PSEH has been reported in the literature, in a patient receiving dual antiplatelet therapy. In the present study, we describe a case of recurrent symptomatic spinal epidural hematoma after cervical spinal surgery in a patient who did not have a bleeding disorder and was not receiving antiplatelet therapy.

## 2. Case Report

A 71-year-old man of normal stature presented with numbness in his left upper extremity and difficulty walking. Physical examination revealed apparent myelopathy and slight weakness of his left hand. Reflexes were symmetric throughout the upper and lower extremities.

**Figure 1:**
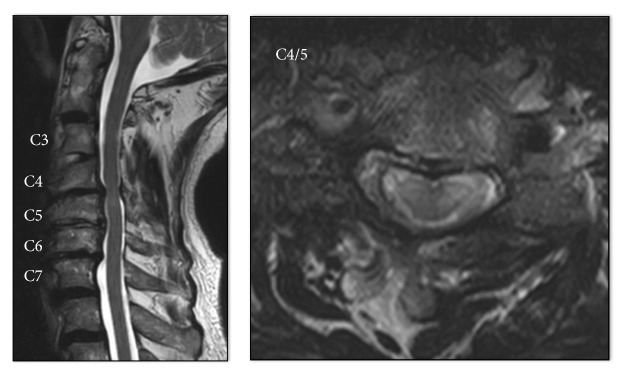
Magnetic resonance image showing C3–C7 degenerative spondylosis with mild central canal stenosis and high-signal lesions at C4/5.

Magnetic resonance imaging (MRI) of the cervical spine showed C3–C7 degenerative spondylosis with mild central canal stenosis and high-signal lesions in the spinal cord (Figure 1). Findings from other examinations were unremarkable. Surgical decompression was advised.

The patient underwent posterior laminectomy at C4 and C5 (subtotal) without instrumentation or fusion. The surgery was uneventful; total surgical time was 1 hour 29 minutes, and estimated blood loss was 70 mL. A suction drain (diameter, 3 mm; Sumitomo Bakelite Company Limited, Akita, Japan) was placed just below the paraspinal muscles.

Routine postoperative examination performed 6 hours after initial surgery revealed severe pain and numbness in the upper extremities, which was immediately followed by tetraplegia. Physical examination showed bilateral muscle weakness 1/5 throughout on upper and lower extremities. Total amount of drainage was 50 mL. Surgical evacuation performed under local anesthesia revealed epidural hematoma. Soon after evacuation, his motor and sensory function greatly improved. Meticulous hemostasis and closure were performed in the operating room at 2 hours after evacuation; in this case we used a same diameter suction drain. His motor function improved to 4/5 throughout.

Two hours after wound closure, the patient again developed tetraplegia, with the same clinical course. Total amount of drainage after the first evacuation was 120 mL. We again performed surgical evacuation and subsequent wound closure using a thicker (diameter, 5 mm) suction drain placed below the paraspinal muscles, after which his clinical course was uneventful. Total amount of drainage after the second evacuation was 200 mL and removed on the fourth postoperative day. His systolic blood pressure was about 130 mmHg and remained stable throughout the perioperative period. Congenital Protein S deficiency was diagnosed postoperatively. At the final follow-up examination, he had no neurological deficits except for slight numbness in his left upper extremity.

## 3. Discussion

The present case demonstrates that PSEH can occur repeatedly and that both bleeding disorders and hypercoagulability can lead to symptomatic PSEH.

Previous studies showed that the incidence of PSEH ranges from 0.1% to 0.4% and is usually less than 1% [1–5]. Recurrence of PSEH is even rarer. Caruso et al. reported a case of recurrent PSEH that occurred after spinal surgery [6]. The patient developed two hematomas, 20 and 37 days after initial surgery. He had a history of obstructive pulmonary disease and acute heart attack and was thus receiving dual antiplatelet therapy (aspirin and clopidogrel). The authors suggested that the hematomas in their patient should be classified as intermediate between the spontaneous and traumatic forms of hematoma. The present patient had no history of bleeding or coagulopathy disorder and was not receiving antiplatelet or anticoagulant therapy.

Previous reports identified multilevel procedures, excessive blood loss, and preoperative coagulopathy as risk factors for PSEH [2, 3]. In an analysis of 14,932 spinal surgeries, Awad et al. reported that risk factors for PSEH were age over 60 years, preoperative administration of nonsteroidal anti-inflammatory drugs, Rh-positive blood type, multiple operative levels, a hemoglobin level of <10 g/dL, intraoperative blood loss >1 L, and an international normalized ratio >2.0 within the first 48 hours [2]. However, they also concluded that well-controlled anticoagulation did not increase the risk of PSEH.

Congenital Protein S deficiency was diagnosed postoperatively in our patient. Protein S is a vitamin K-dependent natural anticoagulant that serves as a cofactor for activated protein C. Abnormalities in Protein S quality and quantity can result in a hypercoagulable state. This deficiency is a confirmed risk factor of deep venous thrombosis (DVT) which occurs infrequently yet is a leading cause of maternal mortality and morbidity and may also be responsible for obstetric complications such as preeclampsia/eclampsia, recurrent fetal loss, and intrauterine fetal restriction [7]. Protein S deficiency is a well-known risk factor for venous thrombosis (more commonly in Asian populations than in whites), which leads to hypercoagulability and, ultimately, various types of thromboembolism [7]. Long duration of bed rest, surgery, pregnancy, advanced age, and malignant tumors is associated with thrombosis formation. Congenital Protein S deficiency has been identified in 1−7.5% of patients with DVT and in 0.07–0.13% general population in whites. On the other hand, people in Asia have higher prevalence both in DVT patients (12.7%) and general population (0.48–0.63%) [7]. We should suspect Protein S deficiency when a patient has PSEH and in addition, when we see patients with recurrent DVT episodes of uncertain cause, especially when they are Asian and surgical procedure has been indicated for them. In such cases, Protein S decrease should be judged preoperatively by its activity.

In the present case, the suction drain was not functioning just before the first episode of PSEH, and hematoma readily formed in the drainage tube after the first surgical evacuation, which may have led to the second episode of symptomatic PSEH. We assume that Protein S deficiency followed by preoperative bed rest and surgical invasiveness led to severe hypercoagulability and subsequent drain obstruction. Surgeons should be aware that recurrent PSEH can develop and that both bleeding disorders and hypercoagulability are risk factors for PSEH.
